# Association between the systemic immune‐inflammation index and outcomes among atrial fibrillation patients with diabetes undergoing radiofrequency catheter ablation

**DOI:** 10.1002/clc.24116

**Published:** 2023-08-08

**Authors:** Zhihao Zhao, Baoping Jiang, Fengyun Zhang, Ruicong Ma, Xiao Han, Chengzong Li, Chaoqun Zhang, Zhirong Wang, Yu Yang

**Affiliations:** ^1^ Department of Cardiology, The Affiliated Xuzhou Municipal Hospital of Xuzhou Medical University Xuzhou First People's Hospital Xuzhou China; ^2^ Department of Cardiology The Affiliated Hospital of Xuzhou Medical University Xuzhou China; ^3^ Department of Clinical Nutrition The Affiliated Hospital of Xuzhou Medical University Xuzhou China

**Keywords:** atrial fibrillation, diabetes mellitus, radiofrequency catheter ablation, recurrence, systemic immune‐inflammation index

## Abstract

**Purpose:**

To investigate the relationship between the incidence of atrial fibrillation (AF) recurrence and the levels of the systemic immune‐inflammatory index (SII, platelet × neutrophil/lymphocyte ratio) in patients with AF and diabetes mellitus (DM) undergoing after radiofrequency catheter ablation (RFCA).

**Patients and Methods:**

Preoperative SII levels were determined in AF patients with DM undergoing RFCA. Restricted cubic splines were used to determine the correlation between SII and the risk of AF recurrence. Multivariate‐adjusted logistic regression models were constructed to determine the relationship between SII levels and AF recurrence. The predictive value of the clinical model and combined with the SII index was estimated by the area under the receiver‑operating characteristic curve, net reclassification improvement (NRI), and integrated discrimination improvement (IDI).

**Results:**

A total of 204 patients with AF and DM who underwent RFCA in our hospital were included. Seventy‐seven patients had AF recurred during a mean follow‐up of 20 months. Restricted cubic spline analysis showed that when SII ≥ 444.77 × 10^9^/L, there was a positive correlation with the incidence of AF recurrence. In addition, adding the SII to the predictive model for AF recurrence after RFCA in patients with DM and AF could contribute to an increase in C‐statistics (0.798 vs. 0.749, *p* = .034). After SII was incorporated into the clinical model, the comprehensive discrimination and net reclassification tended to improve (IDI and NRI > 0, *p* < .05).

**Conclusion:**

SII was independently and positively associated with recurrence after the first catheter ablation in patients with DM and AF.

## Graphical abstract

Atrial fibrillation (AF) is the most common cardiac arrhythmia and radiofrequency catheter ablation (RFCA) has been widely used in treatment for AF, but the high rate of recurrence after ablation remains a significant clinical problem. Systemic immune‐inflammatory index is an independent risk predictor of recurrence after radiofrequency ablation in patients with AF and diabetes mellitus (DM). Based on this study, clinicians may provide preventive treatment for patients with AF and DM who will be under RFCA.

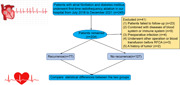

## 1 INTRODUCTION

Recently published data indicate the incidence of diabetes mellitus (DM) combined with atrial fibrillation (AF) has been increasing in recent years.^1^, ^2^ DM patients with AF are associated with a higher risk of heart failure, stroke as well as mortality rates than DM patients with sinus rhythm.^3^ Therefore, it is essential to keep sinus rhythm in DM patients. Radiofrequency catheter ablation (RFCA) superior to antiarrhythmic drugs for AF treatment.^4^ However, AF recurrence remains a major challenge after RFCA, especially in patients with coincidences of AF and DM.^5^, ^6^ Further identifying and controlling the risk factors for AF recurrence may have great clinical importance in developing promising strategies for reducing the incidence of AF recurrence.

Inflammation plays a vital role in the progression of AF.^7^ Patients with DM have higher levels of systemic inflammatory markers.^8^, ^9^ Chronic systemic inflammation can locally increase the risk of atrial myocyte fibrosis, and contribute to the initiation and maintenance of AF.^10^, ^11^ The systemic immune‐inflammation index (neutrophil × platelet/lymphocyte) is a combined inflammatory index and it could serve as a credible and convenient alternative marker for evaluating inflammation in clinical practice.^12^ Also, it is demonstrated that systemic immune‐inflammatory (SII) is significantly associated with an increased risk of atherosclerotic cardiovascular disease.^13^

However, the association between SII and AF recurrence in patients with DM after RFCA remains unknown. Therefore, this study evaluates the relationship between SII and the outcome after RFCA in a cohort of patients with coincidence of AF and DM.

## 2 MATERIALS AND METHODS

### 2.1 Study population

**Figure 1:**
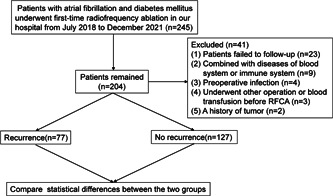
Flow chart of our study.

The flow diagram of our study was shown in Figure 1. Patients who were diagnosed with AF and DM and underwent regular follow‐up after undergoing first‐time radiofrequency ablation from July 2018 to December 2021 in the Affiliated Hospital of Xuzhou Medical University, were reviewed retrospectively. The main exclusion criteria were as follows: (1) preprocedure transthoracic echocardiography, transesophageal echocardiography, or CTA of the left atrial pulmonary vein confirming the presence of left atrial or left atrial appendage mural thrombus; (2) a history of RFCA of AF; (3) severe organic heart disease; (4) congenital visceral disease; (5) patients with recent infections; (6) complex hematologic or rheumatic immune system diseases; (7) a history of the tumor.

### 2.2 Data collection and definitions

Patient demographics and clinical characteristics, including age, sex, body mass index (BMI), smoking history, type of diabetes, type of AF, duration of AF, CHA_2_DS_2_‐VASc score, hypertension, and coronary artery disease were all collected from the electronic medical recording system by trained physicians who were blinded to the aim of the study. In addition, blood markers were recorded including fasting plasma glucose (FBG), glycosylated hemoglobin (HbA1c), serum creatinine, serum uric acid, estimated glomerular filtration rate (eGFR), urea, cystatin C, serum lipid profiles including TG, total cholesterol, low‐density lipoprotein‐C, and high‐density lipoprotein‐C, counts of white blood cells, neutrophils, lymphocytes, platelet, hemoglobin, high sensitivity‐C reactive protein (hs‐CRP). Cardiac ultrasound, 12‐lead electrocardiogram (ECG), and 24‐h ECG were obtained for analysis.

The SII was determined with the following formula: neutrophil × platelet/lymphocyte, the neutrophils to lymphocytes (NLR) index was determined with the following formula: neutrophil/lymphocyte and BMI was calculated as weight (kg)/height squared (m^2^). The diagnosis of DM was confirmed by a history of DM, active treatment with antidiabetic medication, or the typical symptoms of DM with FBG ≥7 mmol/L and/or random blood glucose ≥11.1 mmol/L.

AF recurrence was defined as a documented episode of AF, atrial flutter, or other atrial tachycardia lasting more than 30 s after a 3‐month blank period. Atrial tachycardia that occurred within 3 months did not represent the failure of the operation.

### 2.3 RFCA method

RFCA was performed under the guidance of the CARTO 3 system. The endpoint of ablation was the isolation of the pulmonary veins. Substrate modification of the posterior wall, septum, atrial flutter ablation, and superior vena cava were performed at operator discretion. All patients took amiodarone and rivaroxaban regularly for at least 3 months after the operation.

### 2.4 Follow‐up

Patients were followed‐up regularly after RFCA and 12‐lead ECG and 24‐h ECG were performed at 1, 3, and 6 months. After 6 months, they were followed‐up regularly in the outpatient clinic or by remote telephone. Additional ECG and 24‐h ECG were performed when patients had symptoms of AF.

### 2.5 Statistical analysis

Categorical variables were expressed as counts and percentages (%), while continuous variables were expressed as mean standard deviation or median and interquartile range. To identify determinants of recurrence in AF patients with DM after RFCA, a univariate logistic regression analysis was performed. The baseline variables were selected and included in the multivariable logistic regression analysis model if they showed *p* < .05 in univariate analysis or were clinically relevant to AF recurrence. Restricted cubic spline analyses were used to explore the nonlinear correlation between the SII and the prevalence of AF recurrence. Finally, four models were established to control confounding variables and evaluate the association between the SII and AF recurrence. Model 1 was adjusted for age, sex, and BMI; model 2 was adjusted for variables included in model 1 plus hypertension, smoking, duration of AF, type of AF, CHA_2_DS_2_‐VASc score; model 3, was adjusted for variables in model 2 plus left atrial diameter (LAD), left ventricular ejection fraction (LVEF); and model 4, which is the fully adjusted model, was adjusted for variables in model 3 plus HbA1c, eGFR, hs‐CRP. Furthermore, to evaluate the predictive value of the SII for AF recurrence, the area under the curve (AUC) and the optimal cut‐off value were assessed through receiver operating characteristic (ROC) curve analysis. Moreover, the AUC of NLR was calculated and compared with SII. Meanwhile, to evaluate whether introducing the SII into the model of established risk factors could improve the predictive value, the C‐statistic was calculated and compared by De‐Long's test. Additionally, net reclassification improvement (NRI) and integrated discrimination improvement (IDI) were also calculated to further evaluate the incremental predictive value of the SII index. All statistical analyses were performed using SPSS version 26.0 (SPSS Inc.), and the statistical package R, Version 4.0.3 (https://cran.r-project.org) were prepared. All tests were two‐tailed, and *p* < .05 was considered statistically significant.

## 3 RESULTS

### 3.1 Baseline characteristics

A total of 204 patients who underwent RFCA at a median follow‐up time of 20 months after successful RFCA were enrolled. As shown in Table 1, the mean age of the study population was 55 ± 9 years old, and 161 (78.9%) participants were male. The prevalence of smoking, hypertension, and CAD were 31.3%, 17.2%, and 18.1%, respectively. There were significant differences between the recurrent and non‐recurrent groups in terms of AF type, LAD, LVEF, HbA1c, SII, NLR, neutrophil, and platelet (*p* < .05).

**Table 1:** Baseline characteristics of the study population.

Variable	Total (*n* = 204)	No recurrence (*n* = 127)	Recurrence (*n* = 77)	*Z*/*χ* ^2^/*t*	*p* value
Age (year)	55 ± 9	55 ± 9	55 ± 10	−2.124	.927
Gender				0.007	.935
Male (*n*, %)	161 (78.9)	100 (78.7)	61 (79.2)		
Female (*n*, %)	43 (21.1)	27 (21.3)	16 (20.8)		
Height (m)	1.66 ± 0.08	1.66 ± 0.07	1.65 ± 0.08	−0.013	.990
Weight (kg)	70.40 ± 11.24	69.92 ± 10.33	71.04 ± 12.35	−0.089	.933
BMI (kg/m^2^)	25.39 ± 3.17	25.40 ± 2.89	25.36 ± 3.13	0.106	.916
Comorbidity					
CAD (*n*, %)				1.294	.255
Yes	37 (18.1)	20 (15.7)	17 (22.1)		
No	167 (81.9)	107 (84.3)	60 (77.9)		
Hypertension (*n*, %)				0.091	.762
Yes	35 (17.2)	21 (16.5)	14 (18.2)		
No	169 (82.8)	106 (83.5)	63 (81.8)		
Smoke (*n*, %)				0.130	.719
Yes	64 (31.3)	41 (32.3)	23 (29.9)		
No	139 (68.7)	86 (67.7)	54 (70.1)		
Imaging factors					
LAD (mm)	41 ± 6	40 ± 5	44 ± 6	−5.334	<.001
LVEF (%)	56 ± 6	57 ± 4	54 ± 7	2.954	.004
Laboratory index					
WBC (×10^9^/L)	5.95 ± 1.21	5.85 ± 1.06	6.11 ± 1.43	−1.517	.131
Neutrophil (×10^9^/L)	3.62 ± 1.26	3.35 ± 1.07	4.07 ± 1.43	−4.082	.001
Lymphocyte (×10^9^/L)	1.67 ± 0.58	1.65 ± 0.56	1.71 ± 0.63	−0.701	.484
Monocyte (×10^9^/L)	0.35 ± 0.12	0.34 ± 0.11	0.37 ± 0.14	−1.451	.148
Hemoglobin (g/L)	148 ± 21	149 ± 25	147 ± 12	0.475	.635
Platelet (×10^9^/L)	200 ± 67	188 ± 60	219 ± 74	−3.028	.002
hs‐CRP (mg/L)	2.02 ± 0.47	1.98 ± 0.43	2.11 ± 0.53	−1.956	.052
SCr (µmol/L)	69.55 ± 13.68	68.92 ± 12.81	70.57 ± 15.05	−0.832	.406
SUA (mmol/L)	329.67 ± 101.21	318.94 ± 93.23	347 ± 111.51	−1.873	.063
Urea (µmol/L)	5.50 ± 1.48	5.53 ± 1.37	5.45 ± 1.65	0.364	.728
Cystatin C (mg/L)	0.83 ± 0.14	0.83 ± 0.14	0.84 ± 0.14	−0.211	.883
Triglyceride (mmol/L)	1.57 ± 1.07	1.57 ± 0.57	1.58 ± 1.23	−0.004	.997
TC (mmol/L)	4.30 ± 0.97	4.28 ± 0.92	4.31 ± 1.03	−0.223	.819
HDL‐C (mmol/L)	1.14 ± 0.39	1.15 ± 0.41	1.17 ± 0.33	0.627	.532
LDL‐C (mmol/L)	2.47 ± 0.84	2.42 ± 0.84	2.29 ± 0.84	0.047	.962
FBG (mmol/L)	5.49 ± 1.26	5.47 ± 1.29	5.52 ± 1.20	−0.273	.785
HbA1c (%)	6.82 ± 1.00	6.70 ± 0.97	7.01 ± 0.99	−2.207	.028
eGRF (mL/min/1.73 m^2^)	102.02 ± 16.39	102.35 ± 16.16	101.49 ± 16.86	0.365	.716
NLR	2.37 ± 1.05	2.24 ± 1.12	2.57 ± 0.91	−2.279	.024
SII (×109/L)	461.90 ± 234.73	421.78 ± 222.75	533.17 ± 243.01	−3.345	<.001
Type of AF				16.727	<.001
Paroxysmal (*n*, %)	93 (45.6)	72 (56.7)	21 (27.3)		
Persistent (*n*, %)	111 (54.4)	55 (43.3)	56 (72.7)		
CHA_2_DS_2_‐VASc score	2.2 ± 1.6	2.1 ± 1.6	2.3 ± 1.5	−1.026	.306
AF duration (month)	50 ± 36	46 ± 36	56 ± 34	−1.828	.690

Abbreviations: AF, atrial fibrillation; BMI, body mass index; eGFR, estimated glomerular filtration rate; FBG, fasting plasma glucose; HbA1c, glycosylated hemoglobin; HDL‐C, high‐density lipoprotein‐C; hs‐CRP, high sensitivity‐C reactive protein; LAD, left anterior; LDL‐C, low‐density lipoprotein‐C; LVEF, left ventricular ejection fraction; NLR, neutrophil to lymphocyte ratio; RFCA, radiofrequency catheter ablation; SCr, serum creatinine; SII, systemic immune‐inflammation; SUA, serum uric acid; TC, total cholesterol.

### 3.2 The relationship between SII and the prevalence of AF recurrence after RFCA

**Figure 2:**
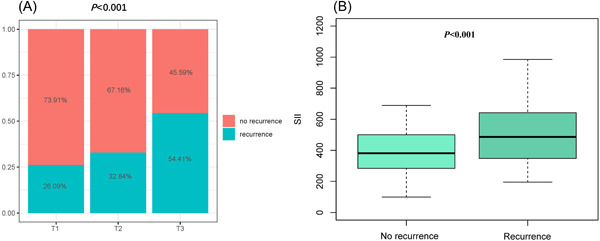
The impact of the SII on the AF recurrence after RFCA (A) and comparison of the SII level between the recurrent group and non‐recurrent groups (B) in the overall study population. AF, atrial fibrillation; RFCA, radiofrequency catheter ablation; SII, systemic immune‐inflammation index.

As shown in Figure 2A, the prevalence of AF recurrence had a stepwise increase with the increasing tertile of the SII index (26.09% vs. 32.84% vs. 54.41%, *p* < .001). Additionally, it is noteworthy that the recurrent group also had a significantly higher SII index than the non‐recurrent group (533.17 ± 243.01 vs. 421.78 ± 222.75, *p* < .001, Figure 2B).

**Figure 3:**
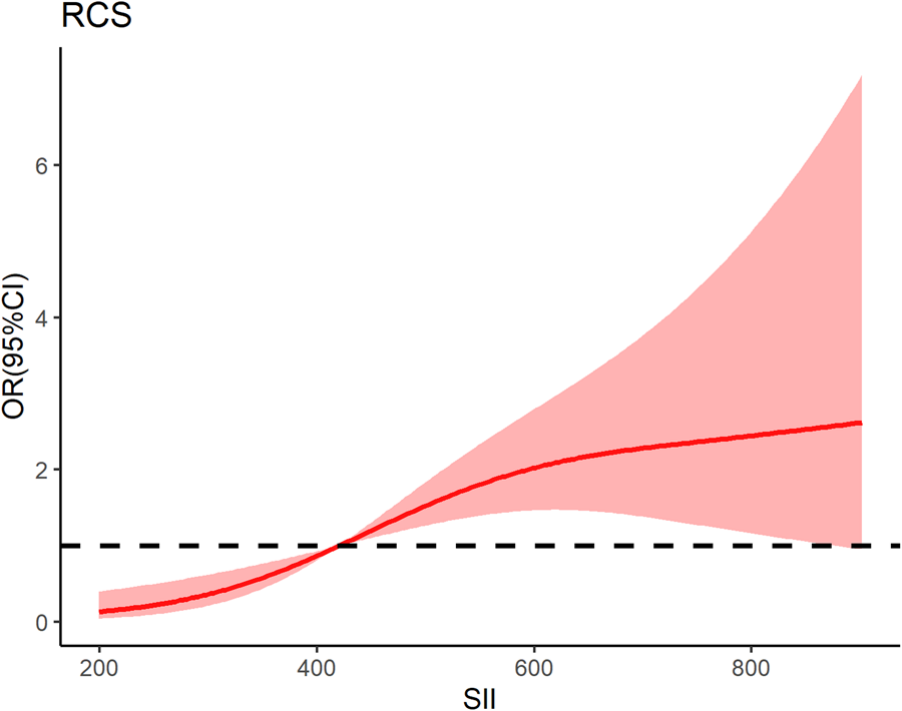
Nonlinear associations between SII concentration and the prevalence of AF recurrence. When the SII concentration was greater than 444.77 × 10^9^/L, the prevalence of AF recurrence increased with SII concentration. When the SII concentration was less than 444.77 × 10^9^/L, the prevalence of AF recurrence reached a plateau. AF, atrial fibrillation; RCS, restricted cubic spline; RFCA, radiofrequency catheter ablation; SII, systemic immune‐inflammation index.

After adjustment for all potential confounders in this study, when the SII was greater than 444.77 × 10^9^/L, the prevalence of AF recurrence increased with SII. When the SII was less than 444.77 × 10^9^/L, the prevalence of AF recurrence reached a plateau (Figure 3).

In multivariable logistic regression models, SII (odds ratio [OR]: 1.328, 95% confidence interval [CI]: 1.153–1.657, *p* < .001) were independent risk factors predicting the AF recurrence after RFCA (Figure S1). Patients with levels of SII above the cut‐off of 444.77 had higher rates of AF recurrence than those with SII levels below 444.77 (21/119 vs. 45/85, *p* < .001), and the association persisted in the four models. In the fully adjusted model 4, the risk of recurrent AF was higher in patients who had SII levels above 444.77 than those with levels below the cut‐off (OR: 3.777, 95% CI: 1.814–7.863, *p* < .001) (Table S1).

### 3.3 Incremental effects of the SII on the predictive value of AF recurrence

**Figure 4:**
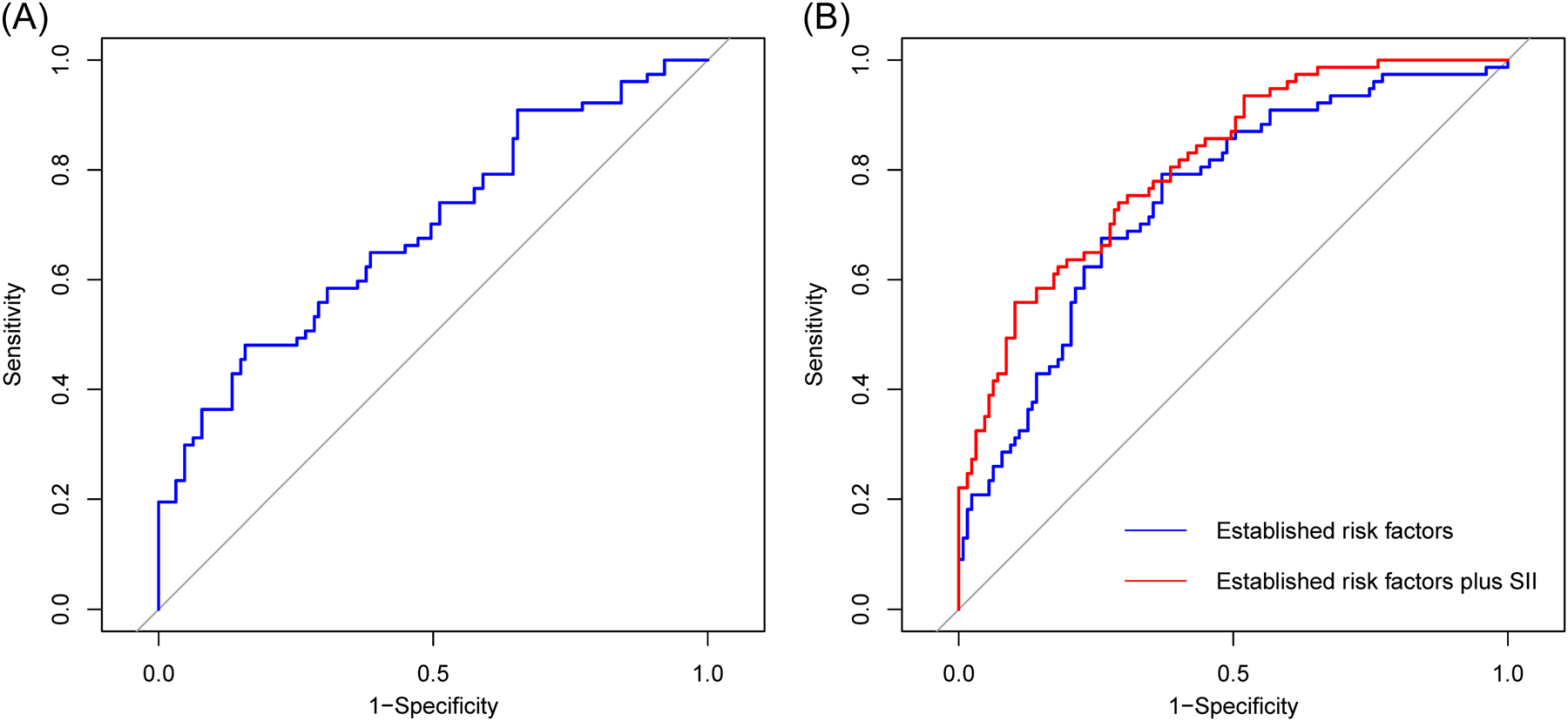
Receiver operating characteristic (ROC) curve analysis of the SII to predict AF recurrence (A) and comparison of the C‐statistics between the models (B). AF, atrial fibrillation; RFCA, radiofrequency catheter ablation; SII, systemic immune‐inflammation index.

As presented in Figure 4A, the ROC curve analysis revealed that the SII could provide a mild predictive value for AF recurrence in patients with DM who had the AUC of 0.684 (95% CI: 0.610−0.758, *p* < .001). The optimal cut‐off value was 444.77 (sensitivity: 61.0%, specificity: 68.5%). Moreover, the AUC of NLR is 0.648 (95% CI: 0.568−0.729, *p* < .001). The optimal cut‐off value was 2.55 (sensitivity: 51.9%, specificity: 81.1%). Meanwhile, the C‐statistic obtained from the model of established risk factors, which consisted of AF type, LAD, and HbA1c, was 0.749 (95% CI: 0.681−0.818, *p* < .001). Furthermore, Table S2 and Figure 4B demonstrate that adding the SII to the model of established risk factors could lead to an increase in C‐statistics (0.798 [95% CI: 0.737–0.859] vs. 0.749 [95% CI: 0.681–0.818], *p* = .034), NRI (0.534 [0.262–0.801], *p* < .001), and IDI (0.078 [0.038−0.118], *p* < .001).

## 4 DISCUSSION

In this comprehensive analysis, we investigate the relationship between inflammation assessed by the SII and AF recurrence in patients with DM undergoing RFCA. The SII was significantly associated with cardiometabolic risk factors. And patients with a higher SII were more likely to have a high risk of AF recurrence after successful RFCA. Besides, SII has a better predictive value than NLR. The SII, either as a continuous or categorical variable, was independently associated with an increased risk of AF recurrence in the fully adjusted model. Besides, LAD, type of AF, and HbA1c were found to be independent risk factors for AF recurrence in patients with DM combined with AF, respectively. In addition, taking the SII into consideration may have clinical significance in the majorization of the early risk stratification of AF recurrence in patients with DM undergoing RFCA.

AF is the most common cardiac arrhythmia worldwide^14^ and DM is the most predominant metabolic disorder in the world.^15^ DM has been described as an important cardiovascular risk factor. Patients with coincidence of AF and DM have a poor quality of life and more severe complications, such as heart failure and stroke.^16^ Currently, RFCA is an effective method for the treatment of AF. Previous studies have shown that many factors, including LAD, glycated hemoglobin, and type of AF, have been reported to be associated with AF recurrence after radiofrequency in patients with DM,^17^, ^18^ which is consistent with our study.

Higher levels of inflammation were found in DM than those in non‐diabetic patients.^19^ A similar observation was found in patients with AF.^20^ Meanwhile, Inflammation participates in the process of myocardial fibrosis.^21^ Accumulating evidence demonstrates that atrial fibrosis is closely associated with AF recurrence.^22^ Direct infiltration of the atrial myocardium by inflammation disrupts the myocardial architecture causing conduction slowing, a significant factor that contributes to the maintenance of electrical remodeling. In addition, cardiac fibroblasts are activated by inflammatory mediators, which contribute to the process of structural remodeling.^11^ The electrical and structural remodeling of the atrium increases susceptibility to AF and promotes the development and maintenance of AF.^23^

The SII can reflect the inflammatory and immune state of the body, which has the characteristics of cheap price and ease of detection in the clinic.^24^ It has been widely applied to different cancers.^25^ In recent years, mounting clinical trials have also been designed to investigate the association the SII with cardiovascular disease.^26^ SII had a better prediction of major cardiovascular events than traditional risk factors in CAD patients after coronary intervention.^27^ SII can predict poor outcomes after elective Off‐Pump CABG.^28^ In addition, SII can predict new‐onset AF after myocardial infarction.^29^ Also, SII could predict the recurrence of AF after cryomaze concomitant with mitral valve surgery.^30^ Besides, SII showed usefulness in predicting AF recurrence after direct current cardioversion.^31^ However, the relationship between SII and AF recurrence after RFCA is still unknown. Neutrophil counts indicate the non‐specific inflammatory response of the organism and reflect a state of subclinical inflammation.^32^ Lymphocytes play an essential role in the regulation of the immune systems^33^ and inflammation enhances the apoptosis of lymphocytes.^34^ NLR has been shown to be significantly associated with the development and recurrence of AF.^35^ In our study, SII was superior to NLR as a prognostic index by comparing the areas under the AUC curve. Platelet index was included in SII additionally compared to NLR. Activated platelets can induce the release of inflammatory substances from endothelial cells and leukocytes.^36^ Therefore, SII can reflect inflammation levels better than NLR.

There are several limitations to this study. First, this was a single‐center retrospective study, and the inherent limitations of such studies inevitably affect patient selection and create selection bias. Second, we calculated SII only once at admission and did not monitor changes in these inflammatory biomarkers during the study period. Also, patients with asymptomatic AF maybe ignored without longer periods of ambulatory monitoring. Third, the cases in this study were from a single source, and the sample size is small, the conclusion needs to be further explored by multicenter and expanded sample size.

Our present work showed that the SII was an independent predictor of AF recurrence in patients with AF and DM. Based on our findings, early evaluation of AF recurrence risk and interventions may be important for the prevention of AF recurrence in the future.

## 5 CONCLUSIONS

Consistent with previous studies, our present study suggested that introducing the SII into a model of established risk factors could improve our ability to identify patients at risk for AF recurrence. Although its incremental predictive value for AF recurrence was limited, considering the large and increasing number of patients with AF and DM admitted for RFCA every year, it still seems to be clinically important to perform assessments of the risk of AF recurrence combined with established risk factors.

## CONFLICT OF INTEREST STATEMENT

The authors declare no conflicts of interest.

## Supporting information

Supporting information.

Supplementary Figure 1: Forest plot of the multivariable logistic regression analysis model in patients with DM exploring the association between SII and AF recurrence after RFCA. Abbreviations: AF, atrial fibrillation; SII, systemic immune‐inflammation index; RFCA, Radiofrequency catheter ablation; BMI, body mass index; LAD, left anterior; LVEF, left ventricular ejection fraction; HbA1c, glycosylated hemoglobin; eGFR, estimated glomerular filtration rate; hs‐CRP, high sensitivity‐C reactive protein; OR, odds ratio; CI, confidence interval.

## Data Availability

The raw data supporting the conclusions of this article will be made available by the authors without undue reservation.
