# Supplementary material for: Association between the systemic immune‐inflammation index and outcomes among atrial fibrillation patients with diabetes undergoing radiofrequency catheter ablation
Source: Clin Cardiol. 2023 Aug 8;46(11):1426–33. doi: 10.1002/clc.24116 (PMC10642337; doi:10.1002/clc.24116)
Supplement: Supplementary file 1 — Supporting information. [file CLC-46-1426-s001.docx]

Supplementary Table 1. Association of SII with AF recurrence following RFCA in multivariable logistic regression models.

| **Variable** | **SII level(×10^9^/L)** | | ***P*-value** |
| --- | --- | --- | --- |
|  | <444.77 | ≥444.77 |  |
| Events/patients | 32/119 | 45/85 |  |
| Incidence (%) | 26.9% | 52.9% |  |
| Model1 | 1.00 (Ref) | 3.089(1.710,5.580) | <0.001 |
| Model2 | 1.00 (Ref) | 2.886(1.543,5.396) | <0.001 |
| Model3 | 1.00 (Ref) | 3.542(1.743,7.198) | <0.001 |
| Model4 | 1.00 (Ref) | 3.777(1.814,7.863) | <0.001 |

**Notes:** Model 1: adjusted for age, sex, and BMI

Model 2: adjusted for age, sex, BMI, hypertension, smoking, AF duration, AF type, CHA2DS2-VASc score

Model 3: adjusted for age, sex, BMI, hypertension, smoking, AF duration, AF type, CHA2DS2-VASc score, LAD, LVEF

Model 4: adjusted for age, sex, BMI, hypertension, smoking, AF duration, AF type, CHA2DS2-VASc score, LAD, LVEF, HbA1c, eGFR, hs-CRP

**Abbreviations**: SII, systemic immune-inflammation; AF, atrial fibrillation; RFCA, Radiofrequency catheter ablation; OR: odds ratio; CI, confidence interval; BMI, body mass index; LAD, left anterior; LVEF, left ventricular ejection fraction; HbA1c, glycosylated hemoglobin; eGFR, estimated glomerular filtration rate; hs-CRP, high sensitivity-C reactive protein; OR, odds ratio; CI, confidence interval.

Supplementary Table 2. Evaluate the predictive power of models for AF recurrence after RFCA

|  | C-statistic | *P*-value | *P* for comparison | NRI | *P*-value | IDI | *P*-value |
| --- | --- | --- | --- | --- | --- | --- | --- |
| Established risk factors | 0.749(0.681- 0.818) | <0.001 | Ref |  | Ref |  | Ref |
| Established risk factors plus SII index | 0.798(0.737-0.859) | <0.001 | 0.034 | 0.534(0.262-0.801) | <0.001 | 0.078(0.038- 0.118) | <0.001 |

**Abbreviations:** SII, systemic immune-inflammation; AF, atrial fibrillation; RFCA, Radiofrequency catheter ablation; NRI, net reclassification improvement; IDI, integrated discrimination improvement.
