# Supplementary figures and images for: Association between the systemic immune‐inflammation index and outcomes among atrial fibrillation patients with diabetes undergoing radiofrequency catheter ablation
Source: Clin Cardiol. 2023 Aug 8;46(11):1426–33. doi: 10.1002/clc.24116 (PMC10642337; doi:10.1002/clc.24116)

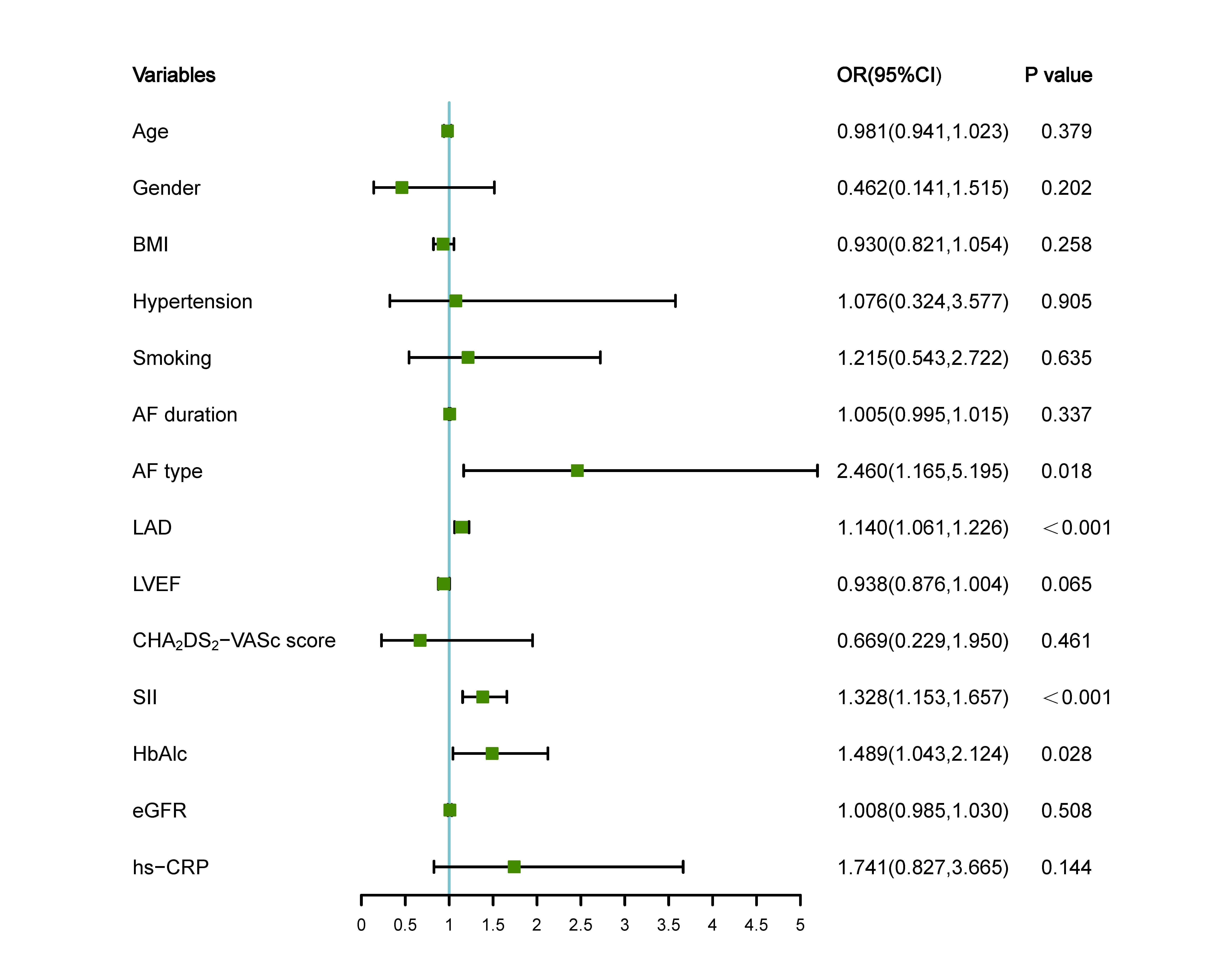

Supplement: Supplementary file 2 — Supplementary Figure 1: Forest plot of the multivariable logistic regression analysis model in patients with DM exploring the association between SII and AF recurrence after RFCA. Abbreviations: AF, atrial fibrillation; SII, systemic immune‐inflammation index; RFCA, Radiofrequency catheter ablation; BMI, body mass index; LAD, left anterior; LVEF, left ventricular ejection fraction; HbA1c, glycosylated hemoglobin; eGFR, estimated glomerular filtration rate; hs‐CRP, high sensitivity‐C reactive protein; OR, odds ratio; CI, confidence interval. [file CLC-46-1426-s002.tiff]
